# Pseudomyxoma peritonei due to low-grade appendiceal mucinous neoplasm with symptoms of inguinal hernia and uterine prolapse: a case report and review of the literature

**DOI:** 10.1007/s13691-017-0297-7

**Published:** 2017-05-13

**Authors:** Hideki Watanabe, Yoshiaki Miyasaka, Kana Watanabe, Ikuko Sakamoto, Hiroshi Nakagomi, Atsushi Takano, Kou Ikegame, Atsushi Yamamoto, Haruka Nakada, Michiya Yasutome, Kazushige Furuya, Masao Hada, Masayuki Inoue, Toshio Oyama

**Affiliations:** 1 Department of Surgery, Yamanashi Prefectural Central Hospital, 1-1-1 Fujimi, Kofu, Yamanashi 400-8506 Japan; 2 Department of Gynecology, Yamanashi Prefectural Central Hospital, Kofu, Japan; 3 Department of Pathology, Yamanashi Prefectural Central Hospital, Kofu, Japan

**Keywords:** Pseudomyxoma peritonei, Low-grade appendiceal mucinous neoplasm, Inguinal hernia, Uterine prolapse

## Abstract

Pseudomyxoma peritonei (PMP) is an unusual condition in which massive amounts of mucinous ascites in conjunction with mucinous peritoneal and omental implants occur. We herein report a case of PMP due to low-grade appendiceal neoplasm (LAMN) and a literature review to clarify the clinical features of PMP. A 68-year-old female suffered from anorexia and abdominal distension and was referred to the emergency department of our hospital. Right-side inguinal hernia and uterine prolapse were revealed by a physical examination. Abdominal computed tomography at admission indicated massive ascites and a ruptured cystic mass in the lower-right abdomen. We diagnosed the patient with a ruptured appendiceal mucinous adenoma and PMP and scheduled a laparotomy. We performed an appendectomy containing the cystic mass, bilateral oophorectomy, and a biopsy for the peritoneum. We irrigated the abdominal cavity using 3000 ml of dextran solution. The macroscopic findings showed a ruptured cystic mass measuring 5 × 4 cm arising from the middle of the appendix. The bilateral ovaries and peritoneum were also covered with yellow mucin. The pathologic findings revealed the presence of low-grade atypical cells inside the capsule. However, no tumor cells were found on the surface of the ovary or peritoneum. A literature review revealed that the prognosis of PMP due to LAMN is relatively good, with a 5-year survival rate of 80%, and hernia is occasionally caused by PMP. According to this literature review, we knew this case might be a typical case. However, PMP is very rare; we need further follow-up data to select an optimal treatment for preventing the relapse of PMP.

## Background

Pseudomyxoma peritonei (PMP) is an unusual condition in which massive amounts of mucinous ascites in conjunction with mucinous peritoneal and omental implants occur [1]. The majority of PMP cases are considered to originate from the appendix and ovary, and occasionally arise from mucinous adenocarcinoma of various organs, such as the colorectum, fallopian tube, gallbladder, urachs, and pancreas [2–5], while in the low-grade or high-grade mucinous neoplasm, the appendix is the primary site in the great majority of cases [1]. Regarding cases with PMP involving both appendix and ovary, the consensus is that the primary site is the appendix, with the ovaries the secondary site, excluding special cases [6–11].

The incidence of PMP is very low; it is estimated to occur in 2 out of 10,000 laparotomies [12]. Therefore, the clinical features have not been well described. Although surgical debulking and appendectomy are considered adequate for initial therapy [1], the etiology, predictive factors of recurrence, and significance of anti-cancer therapies remain to be clarified.

We herein report a case of PMP due to low-grade appendiceal neoplasm and review the literature to clarify the clinical features of PMP.

## Case report

A 68-year-old female was referred to the emergency department of our hospital in April 2016. She was suffering from anorexia, abdominal distension, abdominal pain in lower-right abdomen. Furthermore, a right-side inguinal hernia and uterine prolapse were revealed by a physical examination. Her height and weight were 154 cm and 65 kg, respectively, and she had no history of other diseases.

Laboratory data showed inflammatory changes, as indicated by a white blood cell count of 13,600/μl, CRP of 33.8 mg/dl, hypoalbuminemia at the serum albumin level 3.4 g/dl, and slight renal dysfunction (BUN 108 mg/dl, Creatinine 4.25 mg/dl); in addition, she had elevated levels of tumor markers CEA, CA19-9 and CA125 at 37 ng/ml, 113 U/ml, and 124 U/ml, respectively.

**Fig. 1:**
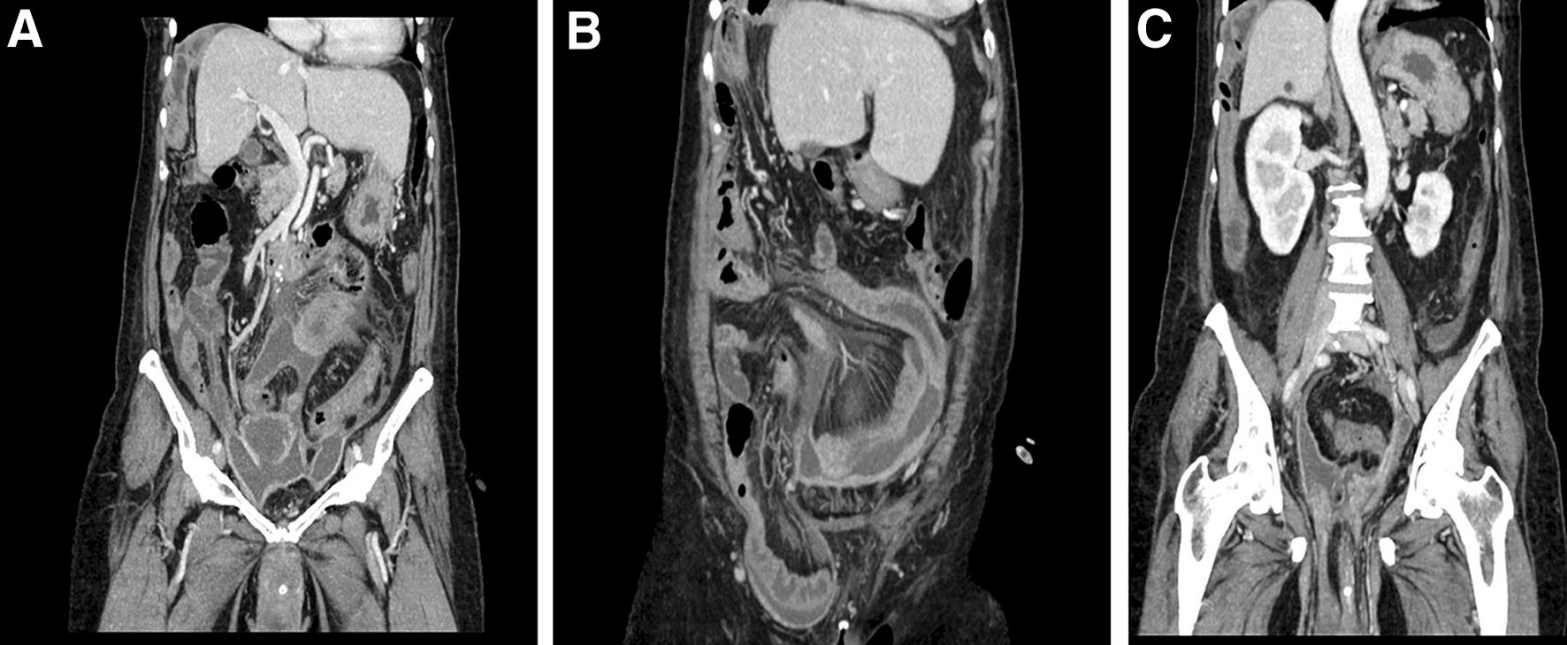
Computed tomography (CT) findings at admission. Coronial view shows massive ascites and a cystic mass in the lower-right abdomen that ruptured to abdominal cavity (**a**). CT also revealed a right inguinal hernia containing the small intestine (**b**) and uterine prolapse (**c**)

**Fig. 2:**
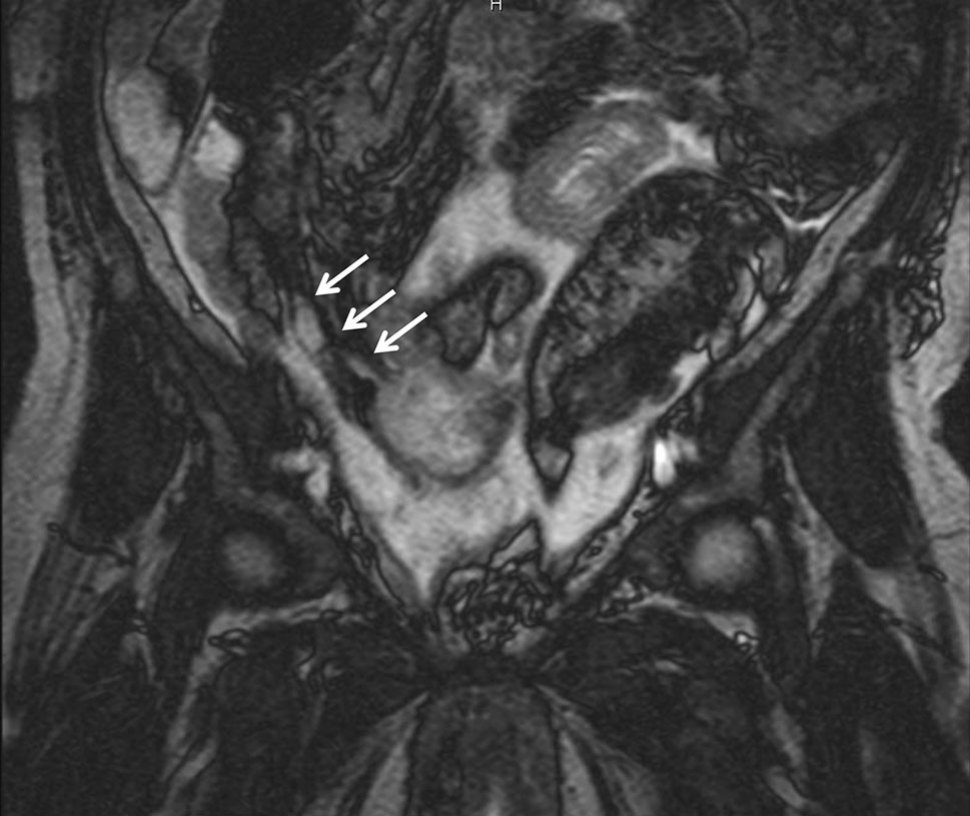
Magnetic resonance imaging (MRI) findings. A cystic tumor is arising from the appendix

**Fig. 3:**
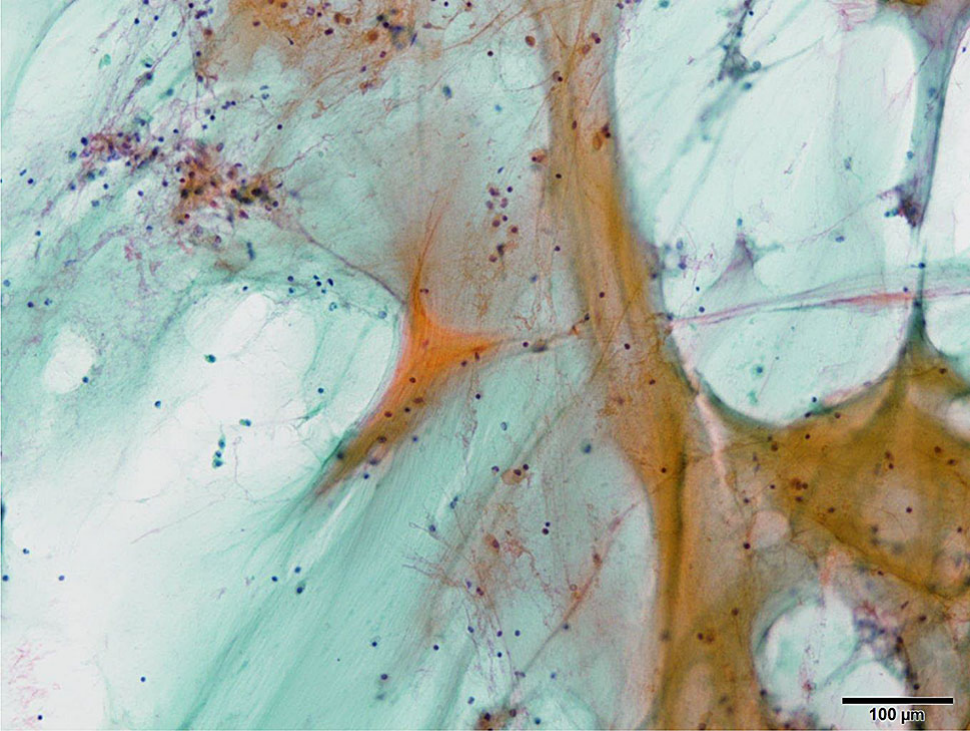
Cytology of the ascites. Mucus indicated the diagnosis of PMP but no malignant cells are seen

Abdominal computed tomography (CT) at admission revealed massive ascites and a cystic mass in the lower-right abdomen that ruptured to abdominal cavity (Fig. 1a). The CT density of the ascites was 10–20 Hounsfield units (H.U.) which was higher than serous ascites (0–5 H.U.) CT also revealed a right inguinal hernia containing the small intestine (Fig. 1b) and uterine prolapse (Fig. 1c). Magnetic resonance imaging revealed that the cystic tumor was arising from appendix (Fig. 2). We had aspirated the ascites being yellow and cloudy. And cytology of the ascites showed mucus suggesting the diagnosis of PMP but no malignant cells (Fig. 3).

**Fig. 4:**
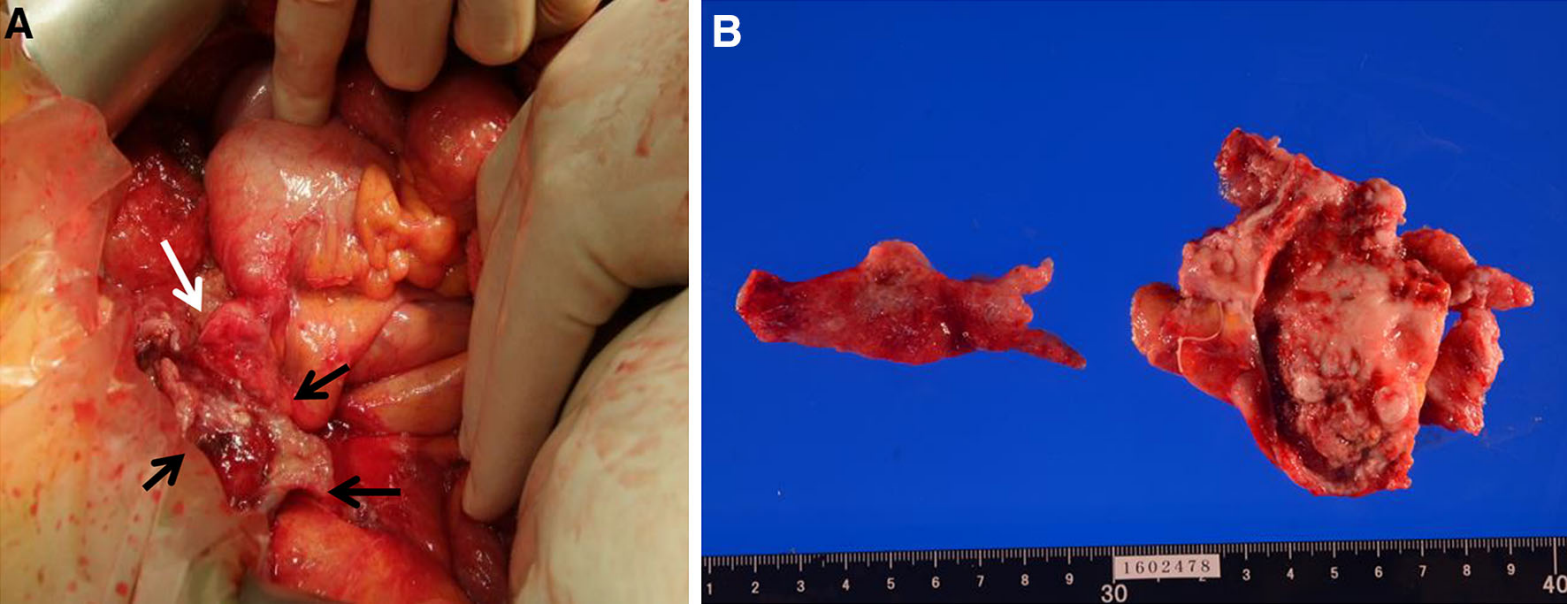
Operative and macroscopic findings of appendix. A ruptured cystic mass measuring 5 × 4 cm is arising from the middle of the appendix (*black arrow*). We also noted small nodules inside the capsule. Mucous is retained in the vermis

Based on these findings, we diagnosed the patient with ruptured appendiceal mucinous adenoma and PMP and scheduled a laparotomy. Massive yellow and cloudy ascites and ruptured cystic tumor arising from the appendix were seen (Fig. 4a). Bilateral ovaries and peritoneum were covered with the yellow substance. We performed an appendectomy containing the cystic mass, bilateral oophorectomy and a biopsy for the peritoneum. We irrigated the abdominal cavity using 3000 ml of dextran solution.

Additionally, we fixed the pelvic diaphragm by sutures and repaired the inguinal hernia via the another incision.

**Fig. 5:**
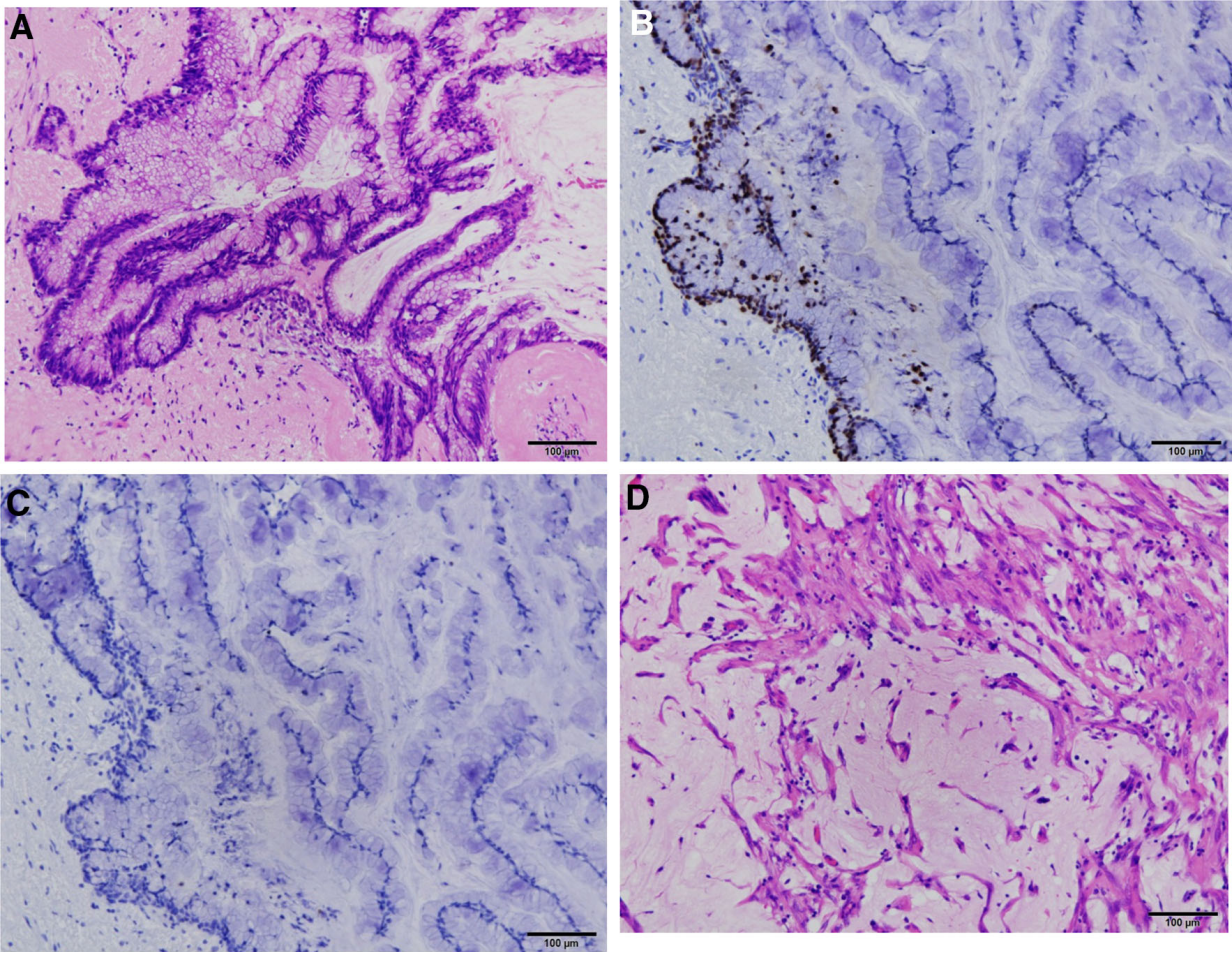
The pathologic findings. HE staining (**a**) shows the presence of low-grade atypical cells inside the capsule which are producing mucin. Immuno-histochemical staining shows high labeling index of Ki 67 (**b**) and no expression of P53 (**c**). While, no tumor cells were found on the surface of the ovary (**d**)

The macroscopic findings showed a ruptured cystic mass measuring 5 × 4 cm arising from the middle of the appendix. We also noted small nodules inside the capsule. Mucous was retained in the vermis. The bilateral ovaries and peritoneum were also covered with yellow mucin. The pathologic findings revealed the presence of low-grade atypical cell inside the capsule, and fibrosis with hemosiderin and cholesterin was observed in the wall of the vermis (Fig. 5) . However, no tumor cells were found on the surface of the ovary or peritoneum.

The postoperative course was uneventful, and the serum tumor marker levels subsequently decreased to CEA 3.1 ng/ml, CA19-9 47.6 U/ml, and CA125 17.7 U/ml, 2 months later.

We administered S1 (75 mg/body for 14 days every 21 days) to prevent the relapse. The patient is doing well in 1 year later.

## Discussion and review of the literature

PMP is a complex disease with unique biological behavior that usually arises from appendiceal mucinous neoplasia. The classification of PMP and its primary appendiceal neoplasm has been discussed and the terminology has changed [13]. In 2010, the WHO proposed the classification of appendiceal mucinous tumors and PMP based on the presence of mucin, and the cytological and architectural features [14]. According to the WHO classification, appendiceal mucinous tumors (excluding signet cell carcinoma) are now divided into two categories: low-grade appendiceal mucinous neoplasm (LAMN) and mucinous adenocarcinoma (MACA). This has been the common classification thus far.

The WHO also defined two categories for PMP as well: low grade and high grade; however, the terms of disseminated peritoneal adenomucinosis (DPAM), peritoneal mucinous carcinoma (PMCA) and discordant type are also allowed. Indeed, PMP with intermediate atypical cells and discordant type is categorized as PMCA-I/D in other reports [13]. Furthermore, it still be controversial whether or not PMP with acellular mucin should be classified separately [13].

The present case was diagnosed as LAMN and acellular-type PMP, and its special clinical features were accompanied by the symptoms of inguinal hernia and uterine prolapse, renal dysfunction and elevated level of tumor markers, such as CEA and CA125.

Adequate follow-up data are required for understanding of the etiology, predictive factors of recurrence, and efficacy of anti-cancer therapies. Therefore, we reviewed 4 cohort studies containing around 100 cases of appendiceal neoplasm with PMP. We also reviewed 15 Japanese case reports with PMP presenting with symptoms of hernia, and anal prolapse, cited in Japan MEDLINE from 1988 to 2014.

For the cohort studies, we reviewed the age at PMP development, gender, pathology, and survival rates. The mean age at the development of PMP was almost the same across all four studies, at around 50 years old (Table 1), with no significant differences observed between LAMN and PMCA (data not shown). Gender differences were observed in only one report [9] that showed 86 females versus 21 males. However, this difference was not seen in the other three reports. Mistrali et al. reported that PMP occurred in 44% (39/88) of LAMN and 50% (8/16) of MACA patients [9]. While, the incidences of DPAM, PMCA-I/D, and PMCA were 60, 18, and 22%, respectively, across the four reports (although the categories of PMCA-I/D differed slightly among these reports).

**Table 1:** Review of the cohort studies for pseudomyxoma due to appendical mucinous neoplasma

Authors (year)	No. of cases	Mean age	Gender		Appendical mucinous tumor			Pseudomyxoma peritonei		
			Male	Female	LAMN	Discordant	MACA	DPAM	PMCA-I/D	PMCA
Ronnett (1995) [6]	109	51	65	44	nd	nd	nd	65	14	30
Misdraji (2003) [9]	107 (50)^a^	54	21	86	88	3	16	39	3	8
Bradley (2006) [6]	101	50	45	56	nd	nd	nd	58	20	23
Guo (2012) [11]	92	53	45	47	nd	nd	nd	49	26	17
Sum of cases	352							211 (60%)	63 (18%)	78 (22%)

*LAMN* low-grade appendiceal mucinous neoplasm, *MACA* mucinous adenocarcinoma, *DPAM* disseminated peritoneal adenomucinosis, *PMCA* peritoneal mucinous carcinomatosis, *-I/D* -intermediate/discordant, *nd* not documented

^a^50 cases developed pseudomyxoma peritonei, among 107 cases with appendicial neoplasm

Regarding the survival, Guo et al. reported that (3-, 5-, 10-year survival rates) of DPMA, PMCA-I/D and PMCA are (97, 80, 65) %, (80, 67, 28) %, and (67, 50, 14) %, respectively [11]. The prognosis of PMCA was significantly worse than that of DPMA and PMCA-I/D. Several authors have described a good prognosis of ruptured LMCA with PMP containing scant epithelial cells just like the present case [13, 15]. These cases were included in the DPMA cases in these cohort studies.

There were 14 cases with symptoms of hernia or prolapse, cited in Japanese MEDLINE from 1988 to 2014: 11 cases of inguinal hernia, one of femoral hernia, umbilical hernia, and anal prolapse (Table 2). The present case developed both inguinal hernia and uterine prolapse (Table 1). The mean age (±SD) was 63 ± 15 years old, and there were far more male cases than females (11 vs 4). Four cases were PMCA, 7 DPMN, 3 acellular mucin and 1 unknown. Serum CEA was elevated more than 5 ng/ml in 3/4 of PMCA and 4/10 of DPMN and acellular mucin. Most of all cases received irrigation and additional therapy such as intraperitoneal administration of anti-cancer drugs, OK432, Mitomycin C, Cisplatin, dextran solution, 5% glucose. Although the effect of hyperthermic intraperitoneal chemotherapy (HIPEC) for PMP [16] and new therapeutic effort was reported for MACA [17], we have no evidences in primary chemotherapy for LMCA. Considering the good prognosis of LMCA, systemic chemotherapy could be reserved for patients with proven recurrence. However, we have no guidelines for systemic therapies for PMP; further follow-up data are necessary to select the optimal treatment.

**Table 2:** Literature review of 15 cases with PMP developed the symptom of hernia or etc

No.	Authors	Year	Gender	Age	Site of hernia and prolapse	Histology		CEA (ng/ml)	Additional therapy
						Appendiceal tumor	PMP		
1	Imai [18]	1988	Male	53	rt ing	LAMN	DPMN	1.4	OK432 ip/UFT po
2	Yamamoto [19]	1989	Male	62	lt ing	LAMN	DPMN	13.2	MMC ip/Tegafur po
3	Matsuda [20]	1996	Male	55	rt ing	LAMN	acellular mucin	nd	nd
4	Iguchi [21]	1999	Male	74	bil ing	LAMN	DPMN	nd	MMC ip
5	Uchida [22]	2000	Female	80	rt fem	LAMN	nd	4.4	nd
6	Yano [23]	2000	Male	31	lt ing	MACA	PMCA	76.6	Dextran/CDDP ip
7	Tanaka [24]	2001	Male	73	rt ing	LAMN	DPMN	9.2	nd
8	Uchiyama [25]	2004	Female	76	Umbilical	MACA	PMCA	12	nd
9	Ohtani [26]	2005	Female	80	Anal prolapse	na	PMCA (ovarian)	27	nd
10	Shinohara [27]	2006	Male	55	lt ing	na	PMCA (urachs)	Normal	Dextran/5’DFUR po
11	Toriguchi [28]	2011	Male	67	rt ing	LAMN	Acellular mucin	2	nd
12	Hamaguchi [29]	2012	Male	70	ing	LAMN	DPMN	9	LV/5FU
13	Kubo [30]	2012	Male	42	lt Ing	LAMN	DPMN	25	dextran
14	Fujita [31]	2014	Male	54	lt ing	LAMN	DPMN	nd	HIPEC
15	Presented case		Female	76	lt ing/uterine prolapse	LAMN	Acellular mucin	37	Dextran/S1po

*rt* Right, *lt* left, *bil* bilateral, *ing* inguinal hernia, fem: femoral hernia, *ip* intraperitoneal administration, *po* oral administration, *nd* not documented, *LAMN* low-grade appendiceal mucinous neoplasm, *MACA* mucinous adenocarcinoma, *DPAM* disseminated peritoneal adenomucinosis, *PMCA* peritoneal mucinous carcinomatosis, *LV* leucovorin, *HIPEC* hyperthermic interperitoneal chemotherapy, *MMC* mytomicyn C, *CDDP* cisplatin, *5FU* fluorouracil, *5′DFUR* doxifluridine, *nd* not documented, *na* not applicable

## Conclusions

We experienced a case of PMP due to LAMN that involved symptoms of right inguinal hernia and uterine prolapse. A literature review showed that the prognosis of PMP due to LAMN is relatively good with a 5-year survival rate of 80%, and hernia is occasionally caused by PMP. However, we need further follow-up data to accurately predict the prognosis of PMP.
